# In Vivo Study on Doxycycline Protective Mechanisms during Myocardial Ischemia Injury in Rats

**DOI:** 10.3390/biomedicines12030634

**Published:** 2024-03-13

**Authors:** Anna Krzywonos-Zawadzka, Agnieszka Olejnik, Grzegorz Sawicki, Iwona Bil-Lula

**Affiliations:** 1 Division of Clinical Chemistry and Laboratory Hematology, Department of Medical Laboratory Diagnostics, Faculty of Pharmacy, Wroclaw Medical University, Borowska 211A, 50-556 Wroclaw, Poland; 2 Department of Anatomy, Physiology and Pharmacology, College of Medicine, University of Saskatchewan, Saskatoon, SK S7N 5E5, Canada

**Keywords:** doxycycline, rat myocardial infraction, LAD, MMPs, MLC1, ischemic heart disease

## Abstract

Background: The fact that during myocardial ischemia/reperfusion (I/R) injury, myosin light chain 1 (MLC1) and troponin I (TnI) are degraded by matrix metalloproteases activity has already been well established in both in vitro and ex vivo studies. However, I/R injury is a complex issue based on several overlapping mechanisms. Increased activity of myosin light chain kinase and nitric oxide synthase due to oxidative stress leads to post-translational modifications of MLC1, thus leading to the increased degradation of these proteins. Methods: Wistar rats were subjected to left anterior descending coronary artery occlusion. To measure the pharmacological effect of doxycycline, transthoracic echocardiography as well as biochemical tests, concentrations of TnI, LDH, MLC1, MMP-2 and MMP-9 were performed. Gelatinize activity and cytotoxicity level were also assessed; Results: I.p., administration of doxycycline before LAD occlusion surgery increased TnI and LDH content in the heart and decreased cytotoxicity. A reduction of MMP-2 and MMP-9 concentration and MMP-2 activity after administration of Doxy was also observed, as well as improvement in echocardiographic parameters just 7 days after surgery. Conclusions: Inhibition of MMPs by doxycycline, in vivo, may serve as a protective agent in future therapy.

## 1. Introduction

The fact that matrix metalloproteinase-2 (MMP-2) can function at a cellular level has introduced a new direction for investigating its role in cardiology [1]. It is established that reactive oxygen species (ROS) alter MMP-2 activity in the heart, and that inhibiting MMP-2 protects the heart from oxidative stress induced by ischemia/reperfusion (I/R) [2]. Excessive production of ONOO^−^ during ischemia/reperfusion can potentially affect MMP-2’s intracellular actions in two ways: either by modifying contractile proteins to become substrates for MMP-2 or by enhancing its activity [3]. MMP-2 contributes to acute cardiac dysfunction by degrading intracellular contractile proteins such as troponin I (TnI), titin, myosin light chains 1 and 2 (MLC1 and 2) in oxidative stress-induced heart injury [4,5]. The production and activity of MMPs in vivo are tightly regulated at various levels [6]. Control of MMPs action in relation to oxidative stress promoted by ischemia–reoxygenation (I/R), is the regulation of proteolytic activity of proteinases (not only MMPs) by the post-translational modifications (PTMs). Sarcomeric substrates for MMP-2 in injured heart, such as TnI, titin, MLC1 and MLC2 modified by nitration, hydroxylation, S-nitrosylation and phosphorylation, render previously proteolysis-resistant proteins to become substrates for MMPs.

Our ex vivo study showed that in ex vivo study, oxidative stress-induced modifications like phosphorylation, S-nitrosylation and nitration of MLC1 and MLC2 triggered by ischemia–reperfusion are associated with the degradation of MLCs by MMP-2. Increased activity of MMP-2 limits heart contractile function by degrading MLCs. Notwithstanding, the mechanism by which the proteolytic degradation of contractile proteins occurs must still be established [6].

Previously, we have shown that in in vitro and ex vivo study (isolated cardiomiocytes and Langendorf perfusion model), administration of doxycycline as MMP-2 and MMP-9 inhibitor protects the heart against consequences of ischemia and reperfusion after acute myocardial infarction [7]. However, myocardial damage after ischemia and reperfusion is different from damage after no-reflow (Langendorf mode) ischemia. This is because there is a sterile injury and an inflammation response that causes more tissue loss. The next step of our study was summarizing the pharmacological protection by tetracycline during ischemia–reperfusion performing on in vivo model of myocardial infarction.

In our presented in vivo study on a rat model of myocardial infarction, we showed the protective effect of doxycycline (i.p., at concentration of 100 µM) on the heart injured during left anterior descending coronary artery occlusion (LAD occlusion). Doxycycline (Doxy) decreased both the level of cytotoxicity generated during I/R and overproduction of MMPs, protecting from cardiac contractile protein’s degradation.

The in vivo study of the ischemia injury rat model was based on our long-term experience, where inhibition of MMPs by Doxy at a dose of 100 µM was used in vitro by employing isolated cardiomyocytes, and subsequently in an ex vivo study using the Ludendorff mode [8,9]. A titration concentration-dependent protocol was used on both models. A dose of 100 µM of Doxycycline was indicated as being effective in inhibiting MMPs, which resulted in the full protection of contractility of cardiomiocytes after ischemia and reperfusion as well as the best recovery of mechanical heart function. Doxycycline dosage was determined based on pharmacokinetic data and previous animal studies [10,11,12].

## 2. Materials and Methods

This investigation conforms to The Guide to the Care and Use of Experimental Animals published by the Polish Ministry of Science and Higher Education. This study was approved by the Ethics Committee for Experiments on Animals at the Ludwik Hirszfeld Institute of Immunology and Experimental Therapy Polish Academy of Sciences, Wroclaw, Poland (no. 115/2017/P1) and performed in accordance with the Guide for the Care and Use of Experimental Animals published by the Polish Ministry of Science and Higher Education.

### 2.1. Experimental Animals and Ethical Statement

Pathogen-free, male 5–7-weeks-old rats (Rattus norvegicus, Wistar (WI) WU Cmd), weighting 300–325 g were used in this study. Animals were obtained from the Mossakowski Medical Research Center, Polish Academy of Sciences, Warsaw, Poland. The utmost possible efforts were made to diminish the suffering of the experimental animals and the number of animals needed for this study. Animals were equally and randomly categorized into groups: aerobic control group (aerobic, sham), acute myocardial I/R injury by LAD occlusion, and acute myocardial I/R injury by LAD occlusion with Doxycycline administration (100 µM, i.p.) 30 min before the surgery (I/R_Doxy_).

### 2.2. Drug Preparation

Analytical grade doxycycline was obtained from Sigma Aldrich (St. Louis, MI, USA). Doxycycline stock solution was firstly dissolved in sterile dd H_2_O, and then diluted with sterile saline to a final concentration immediately before administration (i.p.). Final concentrations of doxycycline, 100 µM, administered 30 min before LAD occlusion surgery were determined based on our previous study [13].

### 2.3. Myocardial Infarction Surgery

Analgosedated rats (1.5% isoflurane (Osotec, KARIZOO in air-oxygen mixture) and buprenorphine 0.1 mg/kg bodyweight) were immobilized on a heating platform ventral side up to maintain the body temperature at 37 ± 0.5 °C. The trachea was cannulated and connected to a small rodent respirator. The tidal volume will be set at 7.4 mL/kg and the respiratory rate at 90 breaths/min with an air–oxygen mixture. Heart rate and respiration were continuously monitored by ECG electrodes. Anesthetized and intubated rats underwent left thoracotomy in the fifth intercostal space. The pericardium was opened to expose the left ventricle of the heart, and then the left anterior descending artery was encircled and ligated under the tip of the left atrial appendage using a 7-0 silk suture. After inducing a myocardial infarction, the muscle and skin were closed in layers, and rats were allowed to recover. Heart function was assessed by echocardiography at 1 week and 2 weeks after each procedure. At 2 weeks, hearts were excised and the ischemic and non-ischemic regions of the left ventricle were dissected, and were then flash frozen in liquid nitrogen for further molecular analyses. Sham-operated rats underwent the same procedure without the LAD ligation and saline i.p., application and served as a sham control.

### 2.4. Transthoracic Echocardiography

Analgosedated rats (1.5% isoflurane buprenorphine and 0.1 mg/kg bodyweight) were immobilized on a heating platform ventral side up to maintain the body temperature at 37 ± 0.5 °C. Rats’ chests were shaved, and pre-warmed ultrasound gel was applied to the area of interest. Transthoracic echocardiography (TTE) was performed on days 0, 7 and 14 after myocardial infarction (MI) using a Vevo 2100 system (Visual Sonics, Toronto, ON, Canada) with a 21-MHz transducer (MS250). The following parameters were determined by tracing end-diastolic and end-systolic areas in longitudinal axis: left ventricle volume (LV), left ventricle ejection fraction (LV EF, %), left ventricle fractional shortening (LV FS, %), left ventricle chamber volume (LV V, μL), stroke volume (SV, µL) and cardiac output (CO, mL/min). Left ventricular wall thickness was measured in M-mode at the mid-ventricular level. To confirm the effectiveness of the left anterior descending coronary artery occlusion procedure, we performed TTE just after the procedure as well as the macroscopic examination of left ventricles using Evans blue and TTC staining.

### 2.5. Evans Blue Followed by 2,3,5-Triphenyltetrazolium Chloride Staining of Hearts

At the end of the perfusion protocol, a heart tissue was stained with 5% Evans Blue. Then, the whole stained hearts were placed at −80 °C for 1 h in order to facilitate sectioning. Sections (1 mm thick) were stained with 1% TTC (30 min, 37 °C) and then washed 3 times, 20 min each time, with formalin for contrast enhancement. The section images were used for the determination of areas of infarct (white/yellow) and area at risk (pink/red).

### 2.6. Hematoxylin/Eosin Staining of Hearts

Fresh heart tissues were fixated in 4% formaldehyde, then dehydrated using alcohol. In the next step, samples were embedded in epon araldite resin, sectioned on an ultramicrotome and stained with eosin/hematoxylin, according to previously described protocols [14].

### 2.7. Preparation of Heart Tissue Homogenate

Frozen heart tissue was fragmented in liquid nitrogen and homogenized mechanically in cold buffer: 50 nM Tris-HCl (pH 7.4) containing 3.1 mM sucrose, 1 nM DTT, 10 µg/mL leupeptin, 10 µg/mL soybean trypsin inhibitor, 2 µg/mL aprotinin and 0.1% Triton X-100. Homogenates were centrifuged at 30K× *g* for 10 min at 4 °C and the supernatants were collected and stored at −80 °C for further analysis. Total protein concentration in tissue homogenates was determined using Bradford-based assay (BioRad, Hercules, CA, USA) and standardized by bovine serum albumin (BSA, Merc-Millipore, Burlington, MA, USA).

### 2.8. Quantitative Measurement of Cardiac Troponin I Protein

Rat Cardiac Troponin I SimpleStep ELISA Kit (ab246529, Abcam, Cambridge, UK) was used to determine a cardiac troponin I protein in the heart, following the manufacturer’s instructions. In a short, a mixture of the captured antibody was labelled with an affinity tag, and a conjugated detector antibody captured an TnI protein accumulated in the heart. Then, the whole complex is immobilized on the 96-well plate through an anti-tag antibody coating the well. The signal developed by TMB catalyzed by HRP is proportional to the amount TnI in the sample.

### 2.9. Measurement of LDH Activity

To determine the activity of lactate dehydrogenase in rat hearts, a Lactate Dehydrogenase Activity Assay Kit (Sigma-Aldrich, St. Louis, MI, USA) was used as we have described previously [2]. Lactate dehydrogenase interconverts pyruvate and lactate with the reduction of NAD to NADH, which is next detected by colorimetric at 450 nm using spark multimode microplate reader *(*Tecan Trading AG, Männedorf, Switzerland). LDH serves as a marker of cell damage due to its cytoplasmic location and release into extracellular space as a result of membrane damage/permeability.

### 2.10. Assessment of Cytotoxicity Level in Rat Hearts

To evaluate an influence of MI injury on rat hearts, we also measured the cytotoxicity level by CytoTox-Glo™ Cytotoxicity Assay (Promega, Madison, WI, USA). We performed the assay according to the manufacturer’s instruction as we described previously [15]. In a short, dead-cell protease cleaves the luminogenic substrate (alanyl-alanyl-phenylalanylaminoluciferin-aminoluciferin), and the liberated aminoluciferin product in presence of Ultra-Glo™ Recombinant Luciferase generates luminescence, which we measured. The intensity of luminescence is proportional to the percentage of cells undergoing cytotoxic stress. Assessment of the number of dead cells was based on the measurement of the extracellular activity of released dead-cell protease in hearts homogenates, normalized to protein concentration, that served as a level of cytotoxicity.

### 2.11. Matrix Metalloproteinase Activity Assay

Gelatin zymography for MMPs activity was performed using our modified protocol [16]. Prior to electrophoretic separation, total protein content in samples (heart homogenates) was measured using Bio-Rad Protein Assay Dye Reagent (Bio-Rad Laboratories, München, Germany). BSA (heat shock fraction, ≥98%, Sigma-Aldrich) was used as the protein standard. Subsequently, heart homogenates were adjusted to the same protein concentration and mixed with 4× Laemmli Sample Buffer (Bio-Rad Laboratories, München, Germany). Finally, samples containing 50 µg of protein were then applied to 10% polyacrylamide gel copolymerized with 2 mg/mL gelatine and 0.1% SDS (denaturing but non-reducing conditions). After electrophoresis, gels were washed with 2.5% Triton X-100 and incubated at 37 °C for 18 h in buffer containing 50 mM Tris-HCl, 5 mM CaCl_2_, 150 mM NaCl and 0.05% NaN_3_. Protein bands were stained using 0.05% Coomassie Brilliant Blue G-250 in a mixture of 30% methanol and 10% acetic acid, and then destained with aqueous solution of 4% methanol, 8% acetic acid until bands were clearly visible. Developed gels were scanned with GS-800 calibrated densitometer (model PowerLook 2100 XL-USB, Bio-Rad Laboratories, München, Germany) and MMP-2 activity was measured using Quantity One v. 4.6.9. software (Bio-Rad Laboratories, München, Germany). Relative MMP activity was expressed in arbitrary units (AU) as activity per microgram of total protein.

### 2.12. Measurement of Total Matrix Metalloproteinase 2 and 9 Concentration in Heart Homogenates

Total MMP-2 and total MMP-9 concentration heart homogenates were measured using quantitative sandwich enzyme immunoassay technique using Quantikine ELISA Kit Rat Total MMP-9 Immunoassay and Total MMP-2 Immunoassay (R&D Systems, Minneapolis, MN, USA), according to the manufacturer’s instruction. Briefly, MMP-2 and MMP-9 from cardiac tissue homogenates were immobilized with monoclonal antibody specific to rat MMP-2 and MMP-9 pre-coated onto a microplate. After washing unbound substances, MMP-2/MMP-9 content in the sample were detected using anti-MMP-2/MMP-9 polyclonal antibody conjugated to horseradish peroxidase (HRP). To develop the colorimetric reaction (540 nm), we used TMB substrate solution. Prior to the test, samples were not diluted.

### 2.13. Measurement of MLC1 Concentration

We used a Rat Myosin Light Chain 3 ELISA Kit (Bioassay Technology Laboratory, Shanghai, China) for quantitative of ventricular isoform of myosin light chain 1. Firstly, captured monoclonal antibodies bounded the MLC1 (ventricular) from cardiac tissue homogenates were pre-coated on a plate. Secondly, the biotin-conjugated antibodies detected the antigens, and avidin-HRP conjugate with TMB as a substrate allowed for complex visualization. The reaction, after termination, was measured with Spark multimode microplate reader (Tecan Trading AG, Switzerland) at 450 nm. Samples were diluted 200 times before testing. Cardiac tissue MLC1 concentration was expressed as µg per mg of total protein in the sample.

### 2.14. Statistical Analysis

The statistical analysis was performed with GraphPad Prism 8 software (GraphPad Software, San Diego, CA, USA; v 8.0.1). After assessment of normality of variance changes, ANOVA or nonparametric Kruskal–Wallis tests were used with the post hoc analysis (Tukey’s or Dunn’s multiple comparisons tests). To assess the correlation between variables, Pearson’s or Spearman’s test was used. Data are presented as boxplots (the mean ± SEM). A probability value *p* < 0.05 was indicated as statistically significant.

## 3. Results

### 3.1. Procedures and Mortality

All sham operated rats and 13 out of 15 I/R and I/R_Doxy_ rats survived throughout the study (aerobic control: n = 3; I/R control group: n = 9; I/R group with Doxy 100 µM treatment: n = 5). Hence, the overall survival rate was 88%. One of the two rats died during recovery from anesthesia, and the second died during coronary artery ligation surgery. The mean age at the time of surgery was 6 ± 0.5 weeks for sham, 6.3 ± 0.5 weeks for I/R and 6.5 ± 0.55 weeks I/R_Doxy_. The mean body weight at the time of surgery was 320 ± 5 g, 315 ± 10 g and 317 ± 5 g for sham, I/R and I/R_Doxy_ rats, respectively.

### 3.2. Doxycycline Protects from Heart Tissue Damage

**Figure 1:**
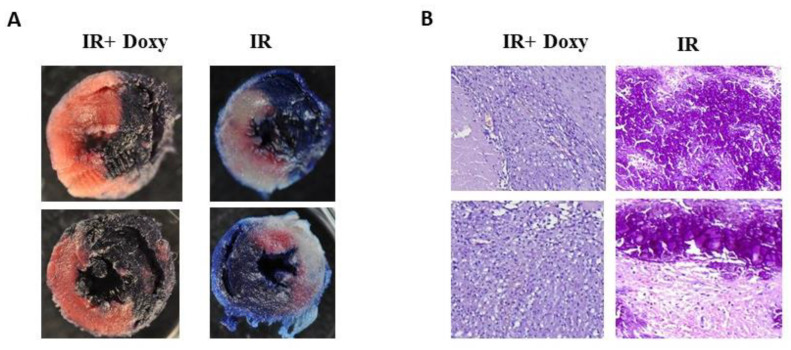
(**A**) Evans Blue followed by 2,3,5-triphenyltetrazolium chloride (TTC) staining of rats’ hearts. Representative heart slices are shown for I/R hearts and IR hearts subjected to doxycycline treatment. White/yellow areas indicated infarct area. (**B**) Hematoxylin/eosin staining of hearts. Representative heart images are shown for I/R hearts and I/R hearts subjected to doxycycline treatment. Magnification 100×.

Macroscopic assessment of infarct size after LAD occlusion, followed by TTC staining of sectioned heart, revealed large areas of infarct (white/yellow), as well as a minor area at risk (pink/red) (Figure 1A). Treatment with Doxy visibly decreased the areas of infarct in favor of the area at risk. Additionally, morphological changes of cardiomyocytes subjected to I/R have been confirmed by H&E staining. Treatment of heart tissue with Doxy maintained normal cardiomyocyte morphology, suggesting a protective effect of the treatment (Figure 1B).

**Figure 2:**
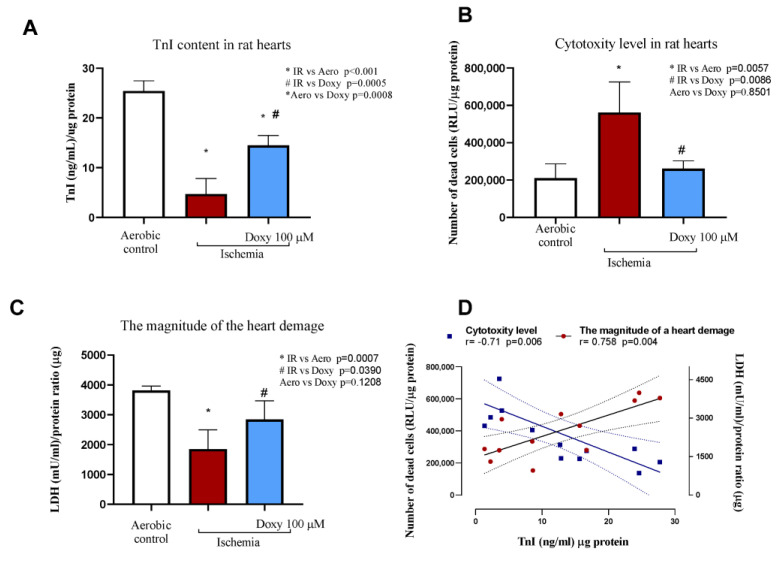
An influence of Doxy on the magnitude of heart ischemia injury: (**A**) Lower TnI concentration in rat hearts served as a marker of damage; (**B**) the number of dead cells in rat hearts based on the activity of dead cell protease, tested by cytotoxicity assay; (**C**) the activity of LDH in hearts as a marker of heart injury. (**D**) Correlation between TnI and other markers of injury: the activity of dead cell protease and LDH. All markers of injury were normalized to protein concentration. mU/mL: milliinternational enzyme units per milliliter; RLU: relative light units; Data are presented as mean ± SEM; n = 3–8; * *p* < 0.05 vs. Aero; # *p* < 0.05 vs. I/R; ANOVA with Tukey’s multiple comparisons tests were used.

As a marker of heart injury after LAD occlusion, we used the number of cardiac dead cells, TnI content in the hearts, as well as activity of LDH. The activity of LDH and TnI content were significantly decreased in hearts that underwent I/R procedure compared to the sham (aerobic control) group. Administration of Doxy100 µM i.p., before MI led to an increase in TnI protein content and LDH activity that were observed before I/R injury (Figure 2A and Figure 1C). After treatment of Doxy, we also observed a decreased number of cardiac dead cells (cytotoxicity level in the heart, Figure 2B) in comparison to the I/R group (*p* = 0.0086). After performing a procedure of LAD occlusion, we observed a strong positive correlation between TnI content in the heart and the magnitude of the heart damage (represented by LDH activity; r = 0.76) and strong negative correlation between TnI and the number of dead cells (r = −0.71) (Figure 2D).

### 3.3. Doxycycline Protects from I/R Associated Impairment of Left Ventricular Function

**Figure 3:**
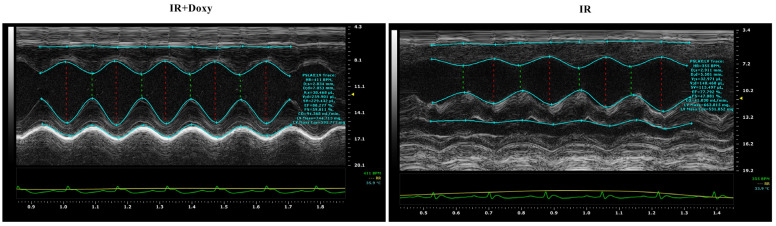
Transthoracic echocardiography images of the rat heart on the 14th day of the experiment, showing representative pictures for both the IR and IR doxy groups.

The echocardiography evaluation demonstrated that the cardiac output (OC, *p* = 0.049), stroke volume (SV, *p* = 0.032), fractional shortening (FS, *p* = 0.018) and ejection fraction (EF, *p* = 0.002) were significantly reduced after I/R, as compared to sham at 7 and 14 days after surgery (Table 1, Figure 3). Also, the end-diastolic and end-systolic volume were also significantly enlarged after I/R (*p* = 0.006 and *p* = 0.033 respectively). However, in the group of animals where the Doxy was administered before LAD occlusion surgery, an improvement in echocardiographic parameters just 7 days after surgery and cardioprotective effect of treatment were observed.

### 3.4. Effect of Doxy on MMPs Activity and Concentration

**Figure 4:**
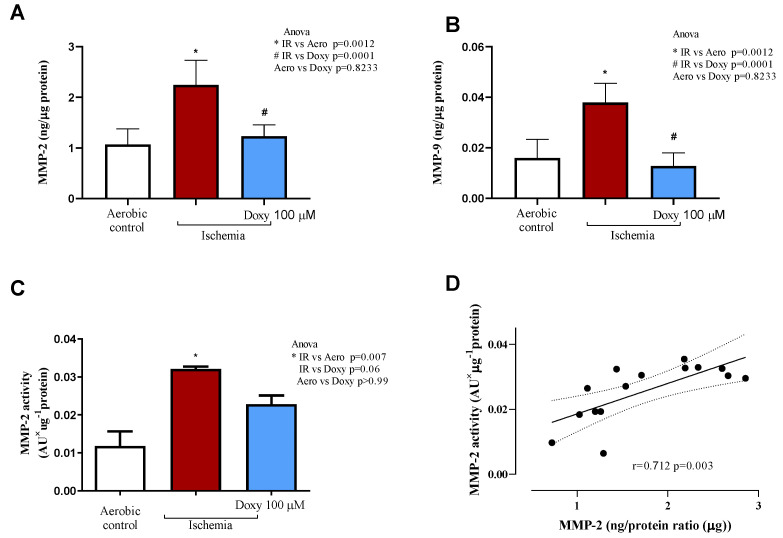
Reduction of MMP-2 and MMP-9 concentration and MMP-2 activity after administration of Doxy 100 µM. MMP-2 (**A**) and MMP-9 (**B**) concentration was normalized to total protein concentration. MMP-2 activity (**C**) was normalized to total protein concentration and expressed in AU. (**D**) Correlation between concentration and activity of MMP-2. MMP, matrix metalloproteinase; * *p* < 0.05 vs. aerobic control; # *p* < 0.05 vs. I/R; n = 3–8.

We observed an increased MMP-2 and MMP-9 concentration (Figure 4A,B) and MMP-2 activity (Figure 4C) in the hearts subjected to I/R. Nevertheless, administration of Doxy 100 µM i.p., 30 min before LAD occlusion surgery normalized both the increased concentration of MMP-2 and MMP-9 in I/R_Doxy_ hearts (Figure 4A,B) and MMP-2 activity to the level observed in the sham group (Figure 4C). Additionally, a strong positive correlation between MMP-2 concentration and activity was observed in all groups (r = 0.712, *p* = 0.03; Figure 4D).

### 3.5. Administration of Doxy Protects from Cardiac Contractile Protein’s Degradation Due to I/R Injury

**Figure 5:**
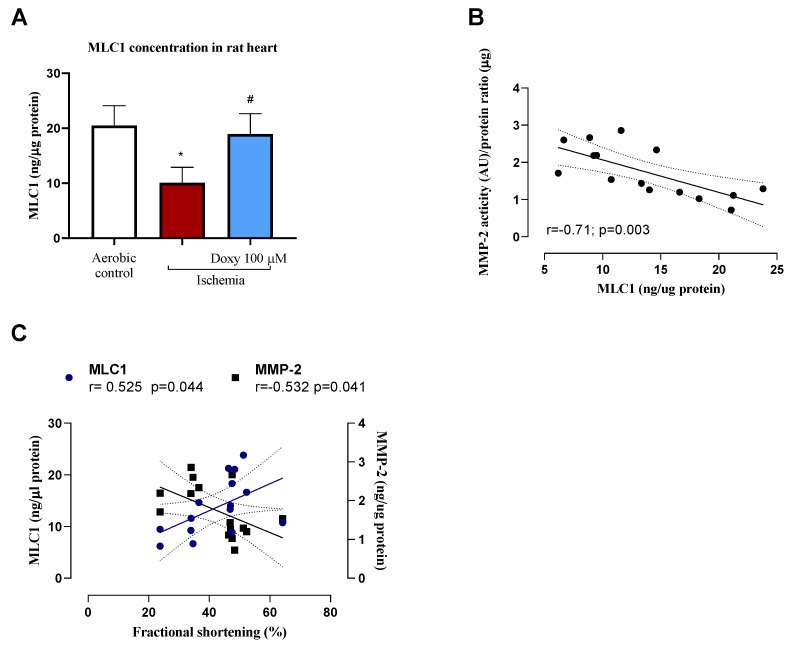
Protective role of Doxy on ventricular MLC1 content in rat hearts (**A**). Correlation of MMP-2 activity and MLC1 tissue content in rat hearts (**B**). Correlation of fractional shortening after 14 days post-surgery (%) and MMP-2 activity and MLC1 tissue content in rat hearts (**C**). MLC1, myosin light chain type 1; * *p* < 0.05 vs. aerobic control; # *p* < 0.05 vs. I/R; n = 3–8.

Administration of Doxy normalized the MLC1 concentration in the heart, and therefore led to decreased tissue injury and ventricular MLC1 degradation to the level observed in a sham group (Figure 5A, *p* = 0.012; I/R vs. Doxy). Degradation of MLC1 in the heart tissue was correlated with increased activity of MMP-2 (Figure 5B; *p* = 0.003; r = −0.71) and fractional shortening after 14 days post-surgery (Figure 5C; *p* = 0.04; r = 0.526).

## 4. Discussion

Cardiovascular diseases, particularly ischemic heart disease and stroke, are the primary cause of global mortality and a significant contributor to a widespread public health issue [17]. Myocardial ischemia reperfusion injury is a clinical consequence characterized by an initial reduction in blood supply, followed by restoration of perfusion and subsequent reoxygenation. This process ultimately results in acute myocardial infarction, severe arrhythmias or heart failure [6]. The primary pathogenesis of myocardial ischemia reperfusion injury is associated with the extensive release of free oxygen radicals, heightened production and activity of metalloproteases, initiation of inflammation cascades, promotion of apoptosis, and myocardial necrosis [18]. As hypoxic injury to the cardiovascular system is a frequent complication following ischemia, comprehensively understanding the mechanisms underlying damage in ischemic heart disease and discovering new effective medications remain the focal points of our research efforts [19]. As cardiovascular hypoxic injury ranks among the most common complications resulting from ischemia, our research focus remains on comprehensively grasping the mechanism underlying damage in ischemic heart disease and discovering novel, effective pharmaceutical interventions [20,21].

In the background of ischemic injury, MMP-2 plays a crucial role in the degradation of extracellular proteins and remodeling of the extracellular matrix. The discovery of the action of MMP-2 was the factor inducing us to verify the current research and support hypothesis on the in vivo model of MI.

Matrix metalloproteinases comprise a family of over 25 structurally-related proteolytic enzymes sharing similar substrate specificity. They play crucial roles in various physiological and pathological processes such as atherosclerosis, restenosis, ischemic heart disease and heart failure [20,22]. In our ex vivo ischemia/reperfusion injury model, we demonstrated that MMP-2 operates not only within the extracellular matrix, but also at the cellular level, exerting its effects within minutes rather than days. MMP-2 is ubiquitous across various cell types and is responsible for the degradation of collagens and other extracellular matrix proteins. Additionally, MMP-2 has been implicated in the breakdown of big-endothelin [23], calcitonin-gene related peptide [24], and monocyte chemoattractant protein [25]. Moreover, MMP-2 contributes to acute cardiac dysfunction through the degradation of intracellular contractile machinery proteins, such as troponin I (TnI), titin, myosin light chains 1 and 2 (MLC1 and 2) in oxidative-stress-induced-heart injury [2,6]. Oxidative stress and overproduction of ONOO^−^ modify the contractile proteins of the heart to serve as a substrate for MMP-2 through post-translational modifications such as nitration, hydroxylation, S-nitrosylation and phosphorylation, thus transforming proteins that were previously resistant to proteolysis to become targets for MMPs. Moreover, overproduction of ONOO^−^ during oxidative stress triggered by ischemia and reperfusion increases the activity of MMP-2 [26]. The fact that matrix metalloproteinases mediated degradation of troponin I (TnI) [27] and ventricular myosin light chains 1 contributes to myocardial ischemia/reperfusion injury has already been well established on both in vitro and ex vivo study. However, ischemia–reperfusion injury is a complex issue, and is based on several overlapping mechanisms [6].

MMP activity undergoes regulation through various mechanisms, including transcriptional control, modulation of mRNA stability, secretion, localization and modulation by specific and nonspecific proteinase inhibitors. Activation of MMPs involves proteolytic cleavage of the N-terminal propeptide by a membrane-type MMP [28] resulting in the removal of the propeptide region and subsequent alteration of the binding between a crucial cysteine thiol residue and the active Zn site. This disruption of the Cys-Zn^2+^ bond can also be prompted by oxidizing agents such as ONOO^−^. In vitro, low concentrations of ONOO^−^ (<10 µM) increased MMP-2 activity, while higher concentrations of ONOO^−^ (>10 µM) decreased MMP-2 activity [26].

Activity of MMPs are regulated by naturally occurring tissue inhibitors of metalloproteinases (TIMPs) [29]. During I/R, extensive production of ONOO^−^ may alter the TIMPs conformation leading in consequences to their inactivation and increased MMP activity [30,31,32]. Synthetic inhibitors of MMPs, such as o-phenanthroline, hydroxamates and the tetracycline class of antibiotics such as doxycycline, share the common characteristic of chelating Zn^2+^ [33].

Previously, we have shown through in vitro and ex vivo studies that the administration of matrix metalloproteinase (MMP-2 and MMP-9) inhibitor doxycycline protects the heart against the consequences of ischemia and reperfusion [2,6,13,15]. The next step of our study was to summarize the pharmacological protection of tetracycline in ischemia–reperfusion performed on an in vivo rat model of myocardial infarction. We evaluated the protection role of doxycycline administered to the heart injured by left anterior descending coronary artery occlusion, and determined its influence on cardiac biochemistry.

Degradation of myosin light chain 1 by gelatinase A during myocardial ischemia–reperfusion and reduced concentration of TnI in the heart tissue has been also demonstrated on our in vivo study, showing the similar mechanism, as was observed in vitro and ex vivo. We hypothesized that in vivo, similar to what was observed in ex vivo research, I/R increases both the phosphorylation and nitration of the crucial residues of MLC1 by involvement of ONOO^−^, and increases its sensitizing susceptibility to degradation by MMP-2 [34,35]. Our findings suggest that phosphorylation of MLC1 could play a crucial role in regulating the intracellular activity of MMP-2 and facilitating the degradation of MLC1. These outcomes bolster previous evidence indicating that post-translational modifications of contractile proteins significantly contribute to the cardiac dysfunction pathology observed during and after ischemic events. We have shown that degradation of MLC1 in the heart tissue was correlated with increased activity of MMP-2 (Figure 4B) and correlated with fractional shortening of the heart 14 days post-surgery (Figure 4C). In the hearts subjected to I/R in the presence of doxycycline, administered i.p., 30 min before LAD occlusion surgery, we observed an improvement in echocardiographic parameters such as cardiac output, stroke volume, fractional shortening, ejection fraction in comparison to sham (aerobic controls, Table 1). Also in this group, the ventricular MLC-1 was protected from degradation (Figure 4). The cardio-protective effect of the treatment in the group of animals treated with Doxy was observed just 7 days after LAD occlusion surgery.

We suggest that heart injury arises from increased degradation of contractile proteins such as MLCs and TnI by gelatinase A (Figure 4C). We also suggest that phosphorylation of myosin light chains could be a significant pathway for controlling the intracellular action of MMP-2 and promoting the degradation of the contractile protein.

## 5. Conclusions

Our study showed a protective effect of MMP-2 and MMP-9 inhibition during in vivo study by decreasing the magnitude of heart ischemic injury and functional disturbances. Taking into account all of the above, we suggest that the inhibition of MMP activity by doxycycline in vivo may serve as a protective agent in future therapy.

## Figures and Tables

**Table 1:** The echocardiographic evaluation of hemodynamic parameters of the heart observed after 7 and 14 days after LAD occlusion surgery. n = 3–8 rats per group. Data are given as mean ± SD. Statistical significance of differences between groups (baseline/7 days/14 days); * *p* < 0.005.

Echocardiographic Parameters		Heart Rate (bpm)	Cardiac Output (mL/min)	Stroke Volume (µL)	Fractional Shortening (%)	Ejection Fraction (%)	Enddiastolic Volume (µL)	Endsystolic Volume (µL)
baseline	sham	386.7 ± 20.2	64.7 ± 12	206 ± 27.4	48.2 ± 5.7	87.1 ± 2.5	176.6 ± 36	36.77 ± 8
	I/R	349.3 ± 30.3	68 ± 12	204.4 ± 21.6	46.2 ± 10.9	79.4 ± 11.5	159.8 ± 46.7	26.77 ± 12.6
	I/R Doxy	384.2 ± 48.6	83.6 ± 31	211.1 ± 55.4	51.32 ± 3.7	83.3 ± 11.8	174.9 ± 42.3	32.49 ± 17.6
7 days	sham	408.7 ± 16.3	80.9 ± 9.7	212.5 ± 30	48.53 ± 5.5	95.11 ± 5.7	176.5 ± 17	38 ± 5.6
	I/R	378.3 ± 35	51.73 ± 15.6	140 ± 25.1	27.56 ± 7.5	63.59 ± 12.9	290 ± 99.8	60.93 ± 13.77
	I/R Doxy	384.4 ± 23	89.24 ± 18.4	201.4 ± 45.2	49.02 ± 12.9	87.69 ± 6	219 ± 54.4	26.03 ± 9.8
14 days	sham	369.3 ±18	87.18 ± 17.5	220.9 ± 29.3	50.64 ± 2	93.21 ± 2	184.7 ± 15	28.78 ±7.7
	I/R	378 ± 34.9	54.71 ± 13.1	111.6 ± 44	35.06 ± 8.8	64.99 ± 12.2	280.3 ± 78	55.7, ± 32.9
	I/R Doxy	391.2 ± 43.5	79.17 ± 11.4	194.6 ± 36.3	50.51 ± 7.6	86.38 ± 5	219.4 ± 54	31.93 ±11.5
*p*	sham vs. I/R	0.328/0.313/>0.99	0.971/0.049 */0.009 *	0.996/0.032 */0.006 *	0.934/0.018 */0.012 *	0.57/0.002 */0.002 *	0.844/0.006 */0.016 *	0.566/0.033 */0.002 *
	sham vs. I/R Doxy	0.995/0.502/0.201	0.446/0.756/0.49	0.983/0.9/0.636	0.864/0.997/0.1	0.882/0.582/0.573	0.999/0.39/0.827	0.907/0.36/0.924
	I/R vs. I/R Doxy	0.266/0.931/0.791	0.427/0.004 */0.037 *	0.947/0.033 */0.012 *	0.556/0.005 */0.004 *	0.812/0.004 */0.004 *	0.826/0.037 */0.020 *	0.767/0.001 */0.001 *

## Data Availability

The data of this study will be available upon request from the reader.
